# Identification of eccDNA in Extracellular Vesicles Derived from Human Dermal Fibroblasts Through Nanopore Sequencing

**DOI:** 10.3390/ijms26094144

**Published:** 2025-04-27

**Authors:** Bianca Simonassi-Paiva, Julia Alves Luz, Julia Hellena Ribeiro, Juliano Coelho da Silveira, Camila Azzolin de Souza, Georgios Joannis Pappas Jr, Juliana Lott de Carvalho, Mark Lynch, Robert Pogue, Neil J. Rowan

**Affiliations:** 1 Faculty of Science & Health, Technological University of the Shannon, Athlone Campus, N37HD68 Athlone, Ireland; a00278507@student.tus.ie (B.S.-P.); mark.lynch@tus.ie (M.L.); 2 Department of Cell Biology, University of Brasilia, Brasilia 70910-900, DF, Brazil; julia.luz@aluno.unb.br (J.A.L.); gpappas@unb.br (G.J.P.J.); 3 Genomic Sciences and Biotechnology Program, Catholic University of Brasília, Brasilia 71966-700, DF, Brazil; julia.ribeiro@a.ucb.br; 4 Department of Veterinary Medicine, School of Animal Sciences and Food Engineering, University of São Paulo, Pirassununga 13635-900, SP, Brazil; julandasilveira@usp.br (J.C.d.S.); camilazzolin@usp.br (C.A.d.S.); 5 Faculty of Medicine, University of Brasilia, Brasilia 70910-900, DF, Brazil; juliana.lott@unb.br

**Keywords:** eccDNA, EV, extracellular vesicles, nanopore sequencing, long-read sequencing, human dermal fibroblasts, bioinformatic pipelines

## Abstract

Extrachromosomal circular DNAs (eccDNAs) are heterogeneous circular DNA molecules derived from genomic DNA, and believed to be involved in intercellular communication and in natural biological processes. Extracellular vesicles (EVs) are membrane-bound particles released from all cells, and have been shown to contain various classes of nucleic acids. EVs can play a role in intercellular communication and may be used as biomarkers. This constitutes the first study to demonstrate that EVs derived from healthy human dermal fibroblasts carry eccDNA. eccDNA from EVs and their corresponding donor cells were isolated and sequenced on the Oxford Nanopore MinIon platform, followed by the identification of potential eccDNAs through four different bioinformatic pipelines, namely ecc_Finder, cyrcular-calling, CReSIL, and Flec. Our main findings demonstrate that EVs derived from human dermal fibroblasts carry eccDNA; there is variability in the number of eccDNAs identified in the same sample through different pipelines; and there is variability in the identified eccDNAs across biological replicates. Additionally, eccDNAs characterized in this research had (a) sequences as small as 306 base pairs and as large as 28,958 base pairs across all samples, (b) uneven chromosomal distribution, and (c) an average of 49.7% of the identified eccDNAs harboring gene fragments. Future implications for this novel research include using this framework method to elucidate factors and conditions that may influence the skin aging process and related biogenesis in human dermal cells.

## 1. Introduction

Extrachromosomal circular DNAs (eccDNAs) are circular DNA molecules derived from genomic DNA, displaying heterogeneity in size and number [1,2]. eccDNAs have been identified in a range of eukaryotic organisms and are believed to harbor functions associated with genetic heterogeneity, amplification of oncogenes, and drug resistance [2]. In humans, they have been associated with cancer [3] and other pathologies, such as type II diabetes [4]. eccDNAs have also been identified in healthy tissues [5] and are believed to play a role in intercellular communication [6], as well as in natural biological processes, such as aging, in both human cell lines [7] and other eukaryotic species [8].

There is still a lack of understanding regarding the biogenesis of eccDNA, but it has been proposed that their formation might be linked to processes such as DNA damage and repair pathways [9], apoptotic DNA fragmentation [10], or the formation of loops during DNA replication. DNA replication-associated loop formation would also explain why eccDNAs would be able to incorporate transposable elements and enhancer or promoter components [11]. It is still unknown whether there are genomic regions that favor the formation of eccDNAs, and it has been suggested that eccDNA formation occurs in a random fashion [10,12].

Extracellular vesicles (EVs) are membrane-bound particles released from all cells, formed by a hydrophilic core and an outer lipid layer [13]. The classes of vesicles are characterized by their size and the presence of known surface markers. Traditionally, studied markers include membrane tetraspanins CD81, CD9, and CD63, but biogenesis markers, such as syntenin-1, have been shown to act as universal markers for extracellular vesicles [14,15]. EVs are released by multiple cell types in both physiological and pathological processes, showing the potential of these particles as biomarkers for pathologies, including cancer [16], vascular aging [17], and others [18]. EVs carry proteins, lipids, and metabolites and have been shown to contain various classes of nucleic acids, including DNA, mRNA, miRNA, and other non-coding RNAs, which seem to participate in EV-mediated cellular communication [19]. To this end, EVs have been credited with facilitating the induction of immune responses [20], cellular differentiation [21], and tissue regeneration [22]. This demonstrates their ability to modulate cellular processes, culminating in their well-studied potential to act as therapeutic agents.

Considering the role of both eccDNA and EVs in cellular communication, as well as the rich nucleic acid content in these vesicles, we hypothesized that EVs derived from healthy cells carry eccDNA. EVs were isolated from three biological replicates of primary human dermal fibroblasts without any known pathologies. eccDNA was then isolated from these vesicles and sequenced on the Oxford Nanopore MinIon platform, a third-generation sequencing technology that generates long reads. eccDNAs were identified in all samples through four different bioinformatic pipelines. To our knowledge, this constitutes the first work demonstrating that EVs derived from human dermal fibroblasts carry eccDNA.

## 2. Results

### 2.1. Extracellular Vesicles Characterization

#### 2.1.1. Nanoparticle Tracking Analysis (NTA) and Transmission Electron Microscopy (TEM)

**Figure 1:**
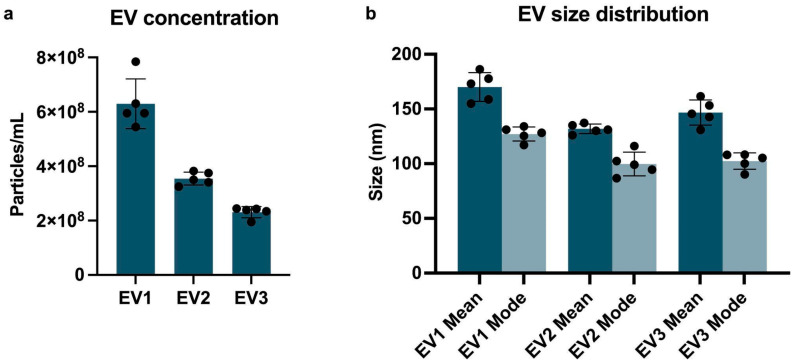
(**a**) Concentration (mean +/− SD) of particles/mL, taken from five different measurements of the same sample. Average concentrations are 6.30^8^ +/− 4.10^7^ particles/mL (EV1), 3.55^8^ +/− 1.06^7^ particles/mL (EV2), and 2.31^8^ +/− 9.08^6^ particles/mL (EV3). (**b**) Average mean and mode (+/− SD) of EVs size distribution (nm): 170.1 nm (EV1 mean); 127.2 (EV1 mode); 131.9 (EV2 mean); 99.8 (EV2 mode); 146.8 (EV3 mean); and 102.4 (EV3 mode). Data obtained from NTA analysis.

**Figure 2:**
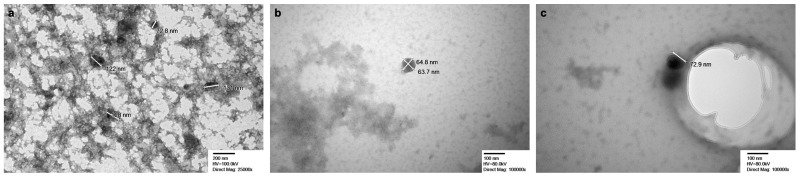
Extracellular vesicles observed under transmission electron microscopy. (**a**) EV1. (**b**) EV2. (**c**) EV3.

Evaluation of EVs by NTA showed average concentration ranges of 6.30 × 10^8^ +/− 4.10 × 10^7^ particles/mL; 3.55 × 10^8^ +/− 1.06 × 10^7^ particles/mL; and 2.31 × 10^8^ +/− 9.08 × 10^6^ particles/mL for EV1, EV2, and EV3, respectively (Figure 1a). Average mode values for particle sizes are 127.2 +/− 2.9 nm (EV1), 99.8 +/− 4.8 (EV2), and 102.4 +/− 3.4 (EV3) (Figure 1b). Supplementary Figure S1 shows the correlation between size distribution and particle concentration. TEM (Figure 2) shows EVs ranging from 64 nm to 189 nm across all three samples.

#### 2.1.2. Flow Cytometry

**Figure 3:**
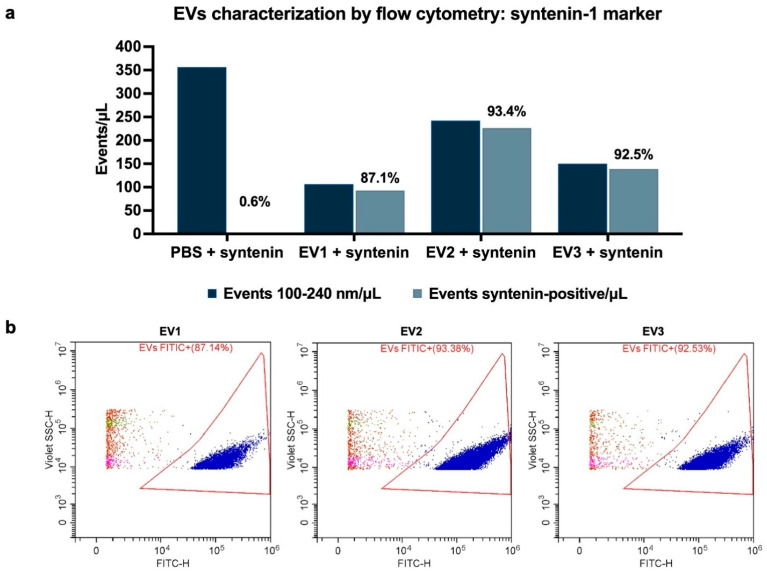
Positive events for syntenin-1 in EV samples. (**a**) Bar graph demonstrating total events/μL, syntenin-1-positive events/μL, and % of positive events in relation to total events in the 100–240 nm gate. (**b**) Flow cytometry dot plots illustrating syntenin-1-positive events in the 100–240 nm gate (indicated in blue) in relation to total events within the 100–240 nm gate (all colors).

EVs were examined for the presence of biogenesis marker syntenin-1 by flow cytometry. Events were gated at the 100–240 nm size range, and the positivity percentage of syntenin was calculated for events within the 100–240 nm gate. 87.1% (EV1), 93.4% (EV2), and 92.5% (EV3) of events were positive for syntenin (Figure 3). The PBS-only control identified only 2.1 events/μL to be positive for syntenin out of 356.65 total events/μL at 100–240 nm, resulting in a positivity rate of 0.6%, indicating that free antibodies or possible artifacts are not interfering with the analysis.

### 2.2. Identification of eccDNAs Isolated from the Extracellular Vesicle Fraction

#### 2.2.1. eccDNAs Are Present in EVs, but There Is Variability Amongst Pipelines in the Identification Process

EV samples isolated from three different primary human adult dermal fibroblasts were individually analyzed with four different pipelines, namely (i) cyrcular-calling, (ii) CReSIL, (iii) ecc_Finder, and (iv) Flec. There was variation in the number of circles identified with each pipeline, with CReSIL and Flec identifying the highest numbers, cyrcular-calling presenting intermediate values, and ecc_Finder consistently acting as the most conservative of the four pipelines used. A similar profile of the pipelines was observed with eccDNAs isolated from matching donor cells (here, D1, D2, and D3).

**Figure 4:**
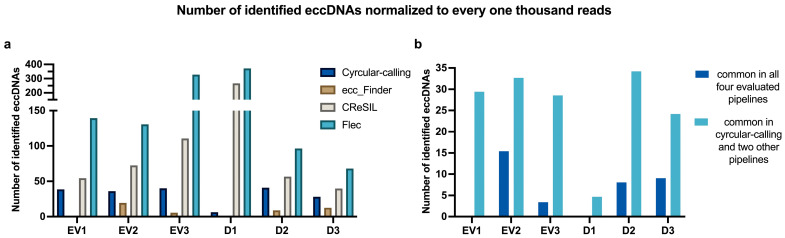
(**a**) Number of eccDNAs identified through each pipeline normalized to every one thousand reads. (**b**) Number of eccDNAs in common on all four pipelines (dark blue) or in cyrcular-calling and any other two pipelines (light blue) normalized to every one thousand reads.

When comparing the outputs of different pipelines, the number of eccDNAs commonly identified by all four pipelines was 0 (EV1); 107 (EV2); 18 (EV3); 0 (D1); 58 (D2); 80 (D3); while the number of consensus circles identified in cyrcular-calling and at least two other pipelines was 45 (EV1); 227 (EV2); 150 (EV3); 18 (D1); 246 (D2); 214 (D3). The latter group (cycrular-calling and two others) comprises the selected circles for profiling of size, chromosomal distribution, and genic regions of overlap. Selecting circles identified by at least three of the four pipelines increases confidence without being overly restrictive. Table 1 highlights the number of eccDNA molecules identified by each pipeline for each sample, and Figure 4 shows the number of eccDNAs normalized to every one thousand reads. Figure 4a highlights individual pipelines, while Figure 4b highlights the normalized number of eccDNAs commonly identified either by all four pipelines or by the cyrcular-calling pipeline and any other two. Table 1 contains raw values of the number of identified eccDNA molecules, whereas Figure 4 normalizes the number of identified eccDNA molecules to every one thousand reads. This approach addresses the overall profiling of eccDNA identification across samples, which is relatively standardized even though the sequencing steps generated a different number of reads in each sample.

#### 2.2.2. Characteristics of Identified eccDNAs: Size, Chromosomal Distribution, GC Content, and Categorization

**Figure 5:**
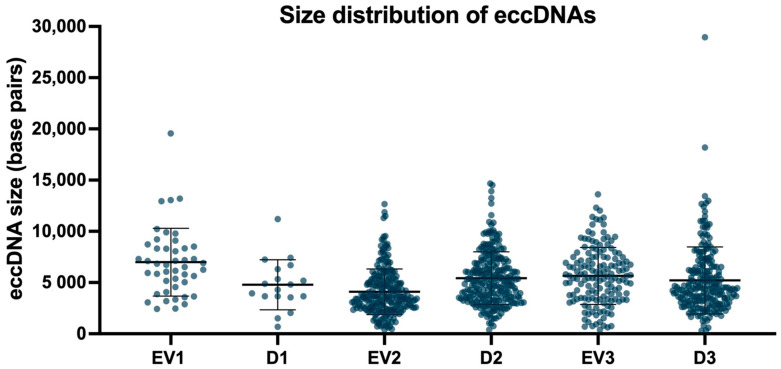
Size distribution (in base pairs) of identified eccDNAs in EV samples and their matching donor cells. Each dot represents an identified eccDNA (individual values). The largest eccDNAs identified are 19,558 bp in EVs (EV1) and 28,958 bp in cells (D3). Mean +/− SD are indicated for each sample.

Variation in the length of identified eccDNAs was observed within and across samples. Across samples, the average size of eccDNAs was 6991 base pairs (bp), 4104 bp, and 5649 bp for EV1, EV2, and EV3, respectively. Within samples, eccDNA sizes ranged from a minimum of 392 bp (EV3) to a maximum of 19,558 bp (EV1). In eccDNAs isolated from cells, the average size of circles was 4784 bp (D1), 5430 bp (D2), and 5209 bp (D3). The smallest eccDNA was 306 bp (D3), and the largest was 28,958 bp (D3). Figure 5 shows the size distribution of identified eccDNAs in EVs and in their matching donor cells.

**Figure 6:**
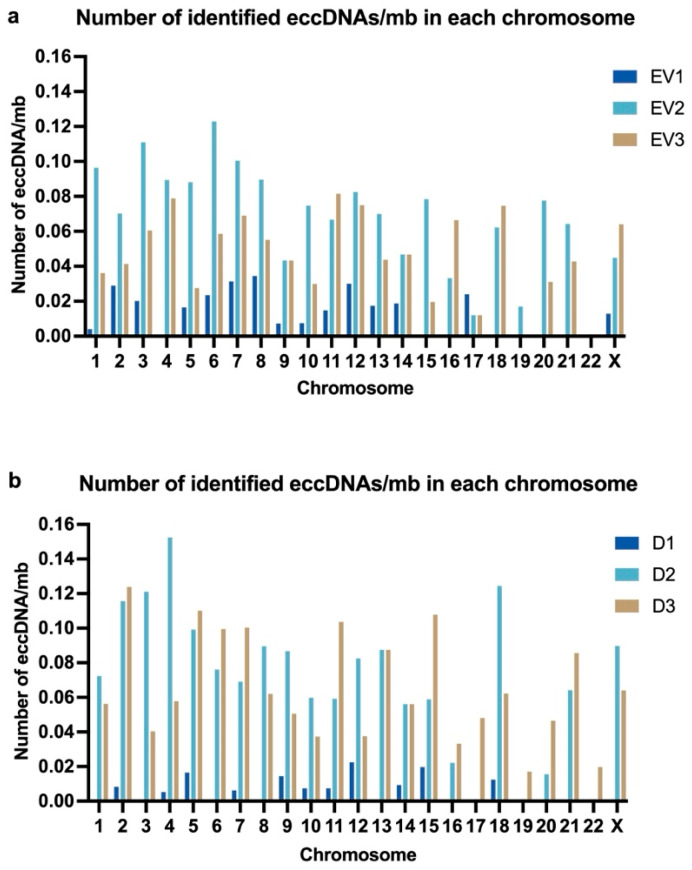
The number of identified eccDNAs in each chromosome for each sample was divided by the size of each chromosome (in megabases), generating an estimated number of eccDNAs/mb/chr. (**a**) eccDNAs/mb/chr in EV samples. (**b**) eccDNAs/mb/chr in cell samples.

Next, we calculated the average number of eccDNAs/mb in each chromosome. eccDNAs were identified in fourteen somatic chromosomes in all three EV samples and in six somatic chromosomes in two EV samples. In one somatic chromosome (chr19), eccDNAs were only identified in one sample (EV2), and no eccDNAs were identified in one chromosome (chr22) (Figure 6a). The graph has no representation of chrY, as cells were derived from female donors; however, eccDNAs were identified in chrX in all three EV samples. There was no overlap of identified eccDNAs across samples. In donor cells’ samples (Figure 6b), eccDNAs were identified in eleven somatic chromosomes in all three samples and in eight somatic chromosomes and in chrX on samples D2 and D3 (but not on D1). In three somatic chromosomes (chr17, chr19, and chr22), eccDNAs were only identified in one sample (D3).

We then selected samples EV1 and D1 to evaluate the GC content surrounding the breakpoint regions of identified eccDNAs, considering 1000 bp upstream and downstream of the breakpoint. In both samples, the GC content varied from 30% to 50%, with the exception of one breakpoint region in EV1 (GC content 25.1%) and one region in D1 (GC content 52.9%). Overall, the average GC % was 37.4% for EV1 and 38.2% for D1. We also evaluated the presence of CpG islands in sequences surrounding the breakpoint for eccDNAs identified on EV1 and D1. For EV1, 10 out of the 45 identified eccDNAs had CpG islands on the 2000 bp sequence surrounding the breakpoint and for D1, CpG islands were observed on 2 of the 18 identified eccDNAs.

**Figure 7:**
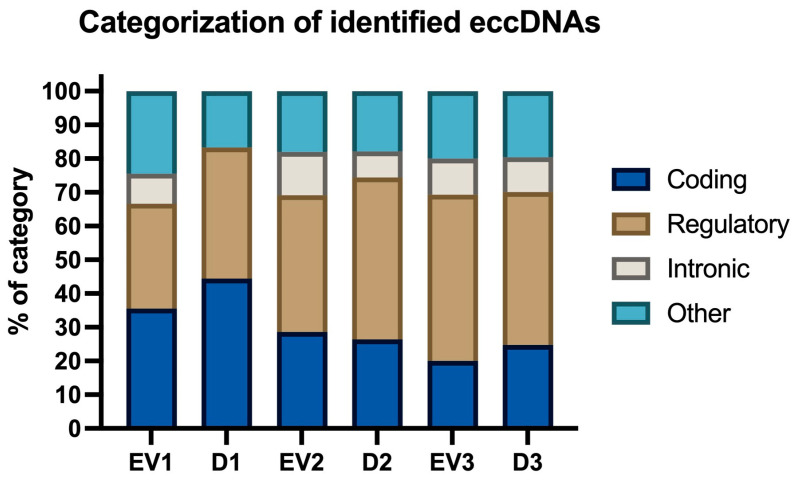
Categorization of eccDNA sequences as coding, regulatory, intronic, or other. Data is indicated as the percentage of eccDNAs in each category, in relation to total eccDNAs in each sample.

Additionally, we evaluated the categorization of sequences, which is a feature of the cyrcular-calling pipeline. “Regulatory” was the most prominent category for four of the samples (EV2, D2, EV3, D3), with a frequency of 40% to 50% occurring in these named samples. In contrast, “coding” was the most prominent category for the samples EV1 and D1, with a frequency of 35% to 45%. It was found that the “intronic” category was the most underrepresented in all samples, with its frequency varying from 0% to 13% (Figure 7).

#### 2.2.3. Gene Fragment-Containing eccDNAs

**Figure 8:**
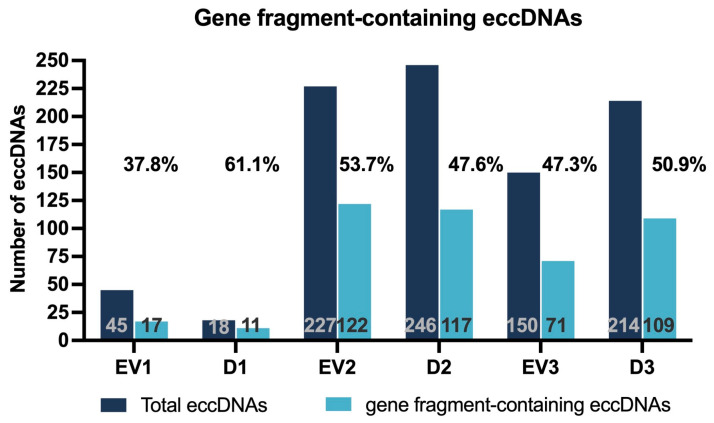
Gene fragment-containing eccDNAs. Numbers in grey inside the dark blue bar indicate the total number of identified eccDNAs for that sample. Numbers in black inside the light blue bar indicate the number of eccDNAs overlapping with genes for that sample. Numbers above the bars indicate the percentage of overlapping eccDNAs in comparison to the total number of eccDNAs for each sample.

In addition to the genomic coordinates of identified eccDNAs, the output of the cyrcular-calling pipeline includes overlapped genes and the breakpoint sequence, accounting for one thousand base pairs before and after the determined breakpoint. Considering only eccDNAs commonly identified in cyrcular-calling and at least two of the other three pipelines, we used the information provided by cyrcular-calling to evaluate the frequency of eccDNAs that overlap with genes (Figure 8). The average of all samples of the percentages of identified eccDNAs harboring gene fragments was 49.7%. For each sample, the percentage of gene fragment-containing eccDNAs in relation to the total number of eccDNAs was 37.8% (EV1), 61.1% (D1), 53.7% (EV2), 47.6% (D2), 47.3% (EV3), and 50.9% (D3). Supplementary Table S1 contains a list of all unique genes overlapped by eccDNAs in each sample.

For the gene ontology analysis, we grouped all the genes identified in all three EV samples, as well as the genes identified in all three samples of donor cells, and they were compared with the Homo sapiens reference list (all genes in the database). No statistically significant results under the “molecular function” category were identified for the list of genes in the EV group, and the only statistically significant term identified under the “molecular function” category for the group of genes in the donor cells group was cell adhesion molecule binding, with a *p*-value of 4.65 × 10^−6^. The top three most significant *p*-values for the “biological process” category are multicellular organism development (2.94 × 10^−7^), anatomical structure development (1.70 × 10^−6^), and system development (2.73 × 10^−6^) for the EV group; and neurogenesis (4.28 × 10^−8^), generation of neurons (1.11 × 10^−7^), and nervous system development (2.61 × 10^−7^) for the donor cells group. In the “cellular component” category, the top three most significant *p*-values are synaptic membrane (8.77 × 10^−10^), neuron projection (1.92 × 10^−8^), and cell projection (8.19 × 10^−8^) for the EV group; and cell junction (1.17 × 10^−11^), synapse (2.32 × 10^−10^), and neuron-to-neuron synapse (9.01 × 10^−7^) for the donor cell group.

#### 2.2.4. eccDNA Similarity in Different Samples

Next, we evaluated the presence of eccDNAs commonly identified across samples based on their genomic coordinates. We compared all EVs, all cells (Ds), as well as each EV sample to its respective cells of origin (e.g., EV1 vs. D1). No similar eccDNA regions were identified in all three EVs nor in all three cells; however, one eccDNA was commonly identified in EV2 and EV3 samples, and a different eccDNA was commonly identified in D2 and D3 samples. When comparing EVs to cells, no similar eccDNAs were identified.

Because different eccDNAs may carry different regions of the same gene, we then checked for similarity in genes harboring eccDNAs between multiple samples. No gene was commonly identified on all three EV samples nor on all three donor samples, but there were six genes in common between EV2 and EV3 (*DACH2*, *LRRC4C*, *GPM6A*, *DAB1*, *PARD6G*, and *GNAI1*); two genes in common between D1 and D3 (*PTPRD* and *GPD2*); and four commonly identified genes between D2 and D3 (*FMN1*, *MDGA2*, *DMD*, and *MLLT10*). Additionally, there were six commonly identified genes between EV2 and D2 (*DACH2*, *KCNMA1*, *PIEZO2*, *GRIP1*, *PTPRM*, and *DACH1*) and two in between EV3 and D3 (*MINDY3* and *CDH13*). Among the gene fragments harboring eccDNAs, there were no genes in common between EV1 and D1.

## 3. Discussion

Extracellular vesicles are known carriers of various nucleic acid species, playing a role in intercellular communication. eccDNAs have been suggested to contribute to genetic heterogeneity and are also believed to be involved in intercellular communication. Thus, we hypothesized and demonstrated that EVs derived from cultured human cells carry eccDNA. We isolated EVs from conditioned media of three primary human dermal fibroblast cell lines and then performed eccDNA isolation, enrichment, and sequencing using the MinIon sequencing platform (Oxford Nanopore). This long-read sequencing technology has been extensively used in the identification of eccDNAs and facilitates the identification of larger eccDNAs in comparison to short-read sequencing [23]. We then analyzed the data through four different pipelines: cyrcular-calling, ecc_Finder, CReSIL, and Flec. The same protocol was carried out for the three primary human dermal fibroblast cell lines from which EVs were isolated.

To our knowledge, this constitutes the first study to identify eccDNA molecules from human dermal fibroblast-derived extracellular vesicles. eccDNAs were identified in all EV samples. However, we observed variability in the number of circles identified in each sample and through different pipelines. Variability in the overall number of eccDNAs per sample was also observed by Ren et al. when working with different isolations of adipose-derived mesenchymal stem cells, where approximately three times more eccDNAs were identified in one isolation of cells compared with isolations of the same cell type from a different donor [7]. Differences in eccDNA counts due to biological variation were also observed in other species, such as pigeons [24]. This observation is similar to what was observed in our work, where there was variability in both the number of reads and the number of eccDNAs identified in each donor sample. Overall, there is a lack of discussion in the literature about the variability of eccDNAs in biological replicates. This can be explained in part by the fact that most studies tend to group their replicates together and evaluate control vs. treatment data only. The variability of eccDNAs amongst same-type cells from different donors may corroborate the hypothesis of a random pattern of formation of eccDNAs [10].

In addition to the variability in the number of identified eccDNAs in each sample, there was minimal overlap of identified circles across samples: of 421 unique eccDNAs identified on EV1, EV2, and EV3, only one was present in EV2 and EV3, and none were commonly identified between EV1 and the other samples. Similarly, of the 477 unique eccDNAs identified in the cells of origin, only one circle was commonly identified between D2 and D3. Dos Santos et al. also observed that identified eccDNAs vary amongst cancer cell line replicates, with recurrence of 0.1% to 0.3% of eccDNAs on two replicates of the same cell line, which was further lowered to 0% to 0.034% when looking for eccDNAs common to three replicates of the same cell line, in both mouse and human cancer cell lines [25]. Similarly, Møller and co-workers observed minimal overlap of identified eccDNAs on different isolations of leukocytes from the same individual [5].

Across all samples, the smallest eccDNA identified was 306 bp, and the largest was 28,958 bp, with average sizes ranging from 4.1 kb to 6.9 kb in different sample groups. Other studies identified the average size of circles varying from 3.5 kb to 4 kb [26], but a myriad of studies also identified the average eccDNA size to be below 1 kb. Although there is great variability across the literature on the average sizes of eccDNAs, this might be related to different sequencing methods, sample origin, or possibly, the random formation pattern of eccDNAs.

The average percentage of the identified eccDNAs harboring gene fragments was 49.7%, with a total of 447 unique genes identified. It is noteworthy that although approximately 2% of the human genome is made up of coding regions of genes, our findings show that almost 50% of the identified eccDNAs harbor gene fragments. The enrichment of eccDNAs in genic regions was also previously reported by Lin et al. [27]. Although further studies are necessary to best explain this, we hypothesize this could be due to a relationship between eccDNA formation and chromatin accessibility, which was also observed by Chen et al. [28], who studied eccDNA formation in single cells. Chen and collaborators observed that eccDNA was enriched in regions marked with H3K4me3 and H3K9me3, histone modifications associated with active enhancer and heterochromatin, suggesting that gene loci, chromatin accessibility, and repair systems influence eccDNA formation [28].

Of the unique genes identified across our samples, 19 (4.44%) were commonly identified in two samples, and only one gene (*DACH2*) was identified in eccDNAs derived from three samples (two EVs and one donor sample). No gene was commonly identified in all three vesicle samples nor in all three donor samples. *DACH2* is located on chr X, associated with premature ovarian failure, and it has been suggested as a biomarker for ovarian cancer [29]. Other noteworthy genes are *GPM6A*, identified in EV2 and EV3, and the PTP receptors (type G identified in D1 and D3; type M identified in EV2 and D2). *GPM6A* expresses a glycoprotein and acts as a signal transducer, among other functions, and has been previously identified in extracellular vesicles [30].

Other published studies have also observed that eccDNAs are distributed across the genome [10,24]. However, there are marked differences in terms of which genomic regions have a greater number of associated eccDNAs. Ren et al. [7] and others [12,31] observed chr19 and chr17 as those with the greater number of eccDNAs/mb. In contrast, we observed that these chromosomes have an overall low number of eccDNAs/mb compared with other chromosomes. Moreover, for chr19, eccDNAs were only identified on EV2 and D3 but were not identified on EV1, EV3, D1, or D2. This may indicate a random pattern of eccDNA formation across the genome, as previously suggested by Wang and collaborators [10].

The uniqueness of our study lies in the use of four different pipelines to identify the eccDNAs, all of which were developed for Nanopore sequencing reads. Still, each pipeline identified different numbers of eccDNAs. While CReSIL and Flec identified the highest numbers of eccDNAs (from 83 to 1725 in different samples), ecc_Finder was the most conservative pipeline, identifying from zero to 136 circles in different samples, and cyrcular-calling was the second-most conservative, identifying from 24 to 295 circles in different samples. Despite this difference in numbers across pipelines, 62% to 90.6% (excluding samples EV1 and D1) of the circles identified on ecc_Finder were also identified on every other pipeline. As for cyrcular-calling, 71.1% to 90.4% of circles were also identified on CReSIL and Flec. These data suggest that there is a validation of most circles identified on more conservative pipelines; however, there is still scope for improvement, such as further development of the bioinformatic profiling of eccDNAs.

There has been a concerted effort to advance our understanding of the potential role of core EV biogenesis in therapies and innovative treatments, including targeting this topic using innovative high-throughput screening strategies [32,33,34]. Extracellular vesicles have been noted to play a critical role in tumor growth and progression through trading between tumor and yonder cells [35], as well as in other pathologies, including neurodegenerative and metabolic diseases [36]. Similarly, although more recently, eccDNAs have also been associated with the pathological evolution of cancer and other diseases [37,38,39]. Given that both EVs and eccDNAs play a potential role in intercellular communication, as well as potential as biomarkers, we explored the presence of eccDNAs as potential molecules in EVs. eccDNAs have been previously identified in EVs derived from plasma, where it was observed that EVs isolated from liver-failure patients carried a greater number of eccDNAs than those from healthy controls [40]. In an opposing manner, our study focused on the isolation of EVs from cultured cells, and the choice of dermal fibroblasts poses an interest in further understanding the relationship between extracellular vesicles and dermal aging.

Potential limitations of this study include the fact that eccDNA was isolated from cells that had been in a serum-free environment for 24 h. This was carried out to ensure eccDNA was isolated from EVs derived from the same culture of cells from which eccDNA was isolated. However, it is appreciated that exposure to different serum-starvation periods may influence eccDNA production by cells. We intend to explore this further in our future research. Additionally, future research will address the study of EV-derived eccDNA for potential interference of cell-free DNA molecules. We also acknowledge the need to explore the relationship between eccDNA formation and methylation patterns. The identification of EV-derived eccDNAs from both short-read and long-read next-generation sequencing methodologies is also merited, as this will contribute to identifying and validating a greater range of circles. Thus, this research will inform many new lines of research inquiry.

## 4. Materials and Methods

### 4.1. Workflow

**Figure 9:**
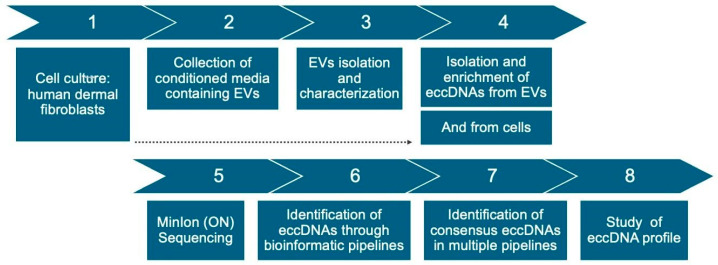
Experimental workflow. Steps 1–3: cell culture and isolation of extracellular vesicles (EVs). Steps 4 and 5: isolation and enrichment of eccDNA from EVs and cells, followed by sequencing. Steps 6–8: bioinformatic analysis for identification of eccDNAs and study of their profile.

This project’s workflow (Figure 9) consisted of culturing three samples of primary human adult fibroblasts and isolating extracellular vesicles from cultured cells’ conditioned media. Then, eccDNA was isolated and enriched from both cells and EVs and sequenced on Oxford Nanopore’s MinIon Platform. Four different bioinformatic pipelines were used for analysis of sequenced data and identification of eccDNAs, and eccDNAs identified in multiple pipelines were selected for a profile study of size, chromosome distribution, and overlapping with gene-containing regions.

### 4.2. Cell Culture

Three lines of primary human adult normal dermal fibroblasts were obtained from PromoCell (Heidelberg, Germany). The cell lines originated from three healthy females aged 34, 27, and 28 years old and were referred to as D1, D2, and D3 throughout this work. Cells were cultured in Dulbecco’s Modified Eagle Medium (low glucose, no phenol red, no L-glutamine; Gibco, Thermo Fisher Scientific, Waltham, MA, USA), supplemented with 1% penicillin-streptomycin, 1% L-glutamine, and 10% Fetal Bovine Serum (FBS), and kept in a humidified incubator at 37 °C and 5% CO_2_. Media changes were performed every 48 h to 72 h, and cells were passaged upon reaching approximately 80% confluency. For the purposes of EV isolation, once cells reached 80% confluency at passage 8, media was removed, cells were washed with phosphate saline buffer (PBS), and fresh serum-free media was added to the cells. This is to avoid the co-isolation of EVs present in FBS. Cells were kept in serum-free media for 24 h, and conditioned media was collected for EV isolation.

### 4.3. Extracellular Vesicle Isolation

Conditioned media was subjected to serial centrifugation steps to remove any remaining cellular debris, as follows: 1000× *g* for 10 min; 2000× *g* for 20 min; and 10,000× *g* for 45 min at 4 °C [41]. Between each sequential centrifugation, the pellet was discarded, and the supernatant was transferred to a clean tube. The remaining media was filtered using a 0.22 μm filter. EV isolation was performed using the ultrafiltration method with Vivaspin^®^20,100 kDa MWCO (Cytiva, Uppsala, Sweden) devices, a protocol adapted from [42]. Devices were prepped with 10 mL of PBS and centrifuged for 10 min at 3000× *g* at 4 °C in a fixed-rotor centrifuge (Mikro 220R, Hettich, Tuttlingen, Germany). Then, conditioned media was subjected to the same centrifugation conditions, adding more media as necessary until all material had been processed. EVs were then resuspended in PBS and stored at −20 °C until used for characterization and DNA isolation. As conditioned media was obtained from three lines of primary human adult dermal fibroblasts, a new device was used for each. Isolated vesicles were named EV1, EV2, and EV3.

### 4.4. Extracellular Vesicle Characterization

EVs were characterized for particle concentration and size by NTA (nanoparticle tracking analysis), transmission electron microscopy (TEM), and flow cytometry for the positive presence of syntenin-1, a marker of EV biogenesis. For NTA, 10 μL of PBS-resuspended EVs were diluted in 990 μL of PBS. The particle size and concentrations were measured using the Nanosight device (NS300; NTA 3.4, build 3.4.003 for EV1 and 3.4.4 for EV2 and EV3, Malvern, UK). Five 30 s videos were taken at a controlled temperature of 38.5 °C and a camera level of 13. A threshold of 5 was considered for the analysis. For TEM, samples were placed on a copper grid and stained with a pioloform-coated solution, followed by 2% aqueous uranyl acetate and drying. Images were acquired using a FEI 200 kV Tecnal 20 LAB_6_ emitter transmission microscope (FEI Company, Hillsboro, OR, USA).

For flow cytometry, 10 μL of each EV sample was incubated with 1 μL of primary and secondary antibodies against syntenin-1. Samples were then diluted in 4 mL of PBS, followed by a second dilution where 50 μL from the first dilution was added to 1900 μL of PBS. Gates were configured using PBS and PBS + antibodies. The selected event population was 100 nm to 240 nm, and the number of positive events for syntenin-1 was gated from this population. For each EV sample, 20 μL of samples were read using a CytoFLEX Flow Cytometer (Beckman Coulter Life Sciences, Indianapolis, IN, USA), with excitation in the FITC channel.

### 4.5. DNA Isolation and eccDNA Enrichment

DNA was isolated using the DNA Plasmid Isolation Kit (T1010, New England Biolabs, Ipswich, MA, USA). Lysis buffer was added to the pellet of cells or to vesicles resuspended in PBS, and the protocol was followed as per the manufacturer’s instructions. Next, isolated DNA was subjected to treatment with 5 units of the endonuclease MssI (pmel) (Thermo Fisher Scientific, Waltham, MA, USA) for 16 h at 37 °C, followed by 10 min at 65 °C for enzyme inactivation. Then, each sample was treated with ATP-dependent Exonuclease V (M0345, New England Biolabs, Ipswich, MA, USA) at 37 °C (48 h treatment for EV samples and 144 h treatment for cell samples). Setup of the initial reaction was performed following the manufacturer’s protocol, and every 24 h the reaction was supplemented with 10 units of exonuclease V and 1 mM of ATP. After 48 h (for EVs) or 144 h (for cells), the enzyme was heat-inactivated at 70 °C for 30 min. The samples were then subjected to rolling circle amplification (RCA) using EquiPhi29™DNA polymerase (ThermoFisher Scientific, Waltham, MA, USA). Briefly, the first reaction was set up with 7 μL of DNA sample in ultrapure water, 1 μL of 10 × EquiPhi29™ buffer, and 2 μL of exo-resistant random primers (500 μM stock) (ThermoFisher Scientific, Waltham, MA, USA). This was incubated at 95 °C for 3 min, followed by 3 min on ice. This was carried on to a second reaction with the addition of 1 μL of EquiPhi29™ buffer, 2 μL of dNTP mix (10 mM each), 0.2 μL of DTT (0.1 M), 5.8 μL of ultrapure water, and 1 μL of 10× EquiPhi29™ polymerase enzyme and incubated at 30 °C for 48 h, followed by heat inactivation at 65 °C for 10 min. The eccDNA enrichment protocol with endonuclease and exonuclease enzymatic treatments followed by RCA was adapted from [5]. After rolling circle amplification, DNA was quantified using a Qubit™ 3 Fluorometer (Invitrogen, ThermoFisher Scientific, Waltham, MA, USA) and carried forward to sequencing library preparation.

### 4.6. Sequencing

Enriched eccDNA was prepared for sequencing following Oxford Nanopore’s LSK-109 and NBD104 preparation protocol and quantified using a Qubit 3 Fluorometer (Invitrogen, ThermoFisher Scientific, Waltham, MA, USA) between each sequential step. Prepped DNA was sequenced using a Spot-ON Flow Cell, R9 version, and the MinIon Mk1B device (Oxford Nanopore Technologies, Oxford, UK), and reads were basecalled using the MiniKnow software (MinKNOW version 23.11.5; subpackages MinKNOW core 5.8.7, Dorado 7.2.13, Bream 7.8.2, and Script Configuration 5.8.6).

### 4.7. Bioinformatic Analysis

Sequences were analyzed for eccDNA identification through four different publicly available pipelines: CReSIL version 1.0.0 (available on https://github.com/visanuwan/cresil (accessed on 11 July 2023)) [43]; cyrcular-calling version 2.0 (available on https://github.com/snakemake-workflows/cyrcular-calling (accessed on 11 July 2023)) [44]; ecc_finder version 1.0.0 (6 October 2021) (available on https://github.com/njaupan/ecc_finder (accessed on 11 July 2023)) [45]; and Flec (eccDNA_RCA_nanopore) version 1.0 (available on https://github.com/YiZhang-lab/eccDNA_RCA_nanopore (accessed on 11 October 2023)) [46].

The reference genome used for mapping was the soft-masked GRCh38.110 downloaded from Ensembl, and RepeatMasker files were obtained from UCSC. All four pipelines were installed in separate conda environments [Anaconda Software Distribution. Computer software. Vers. 23.5.2. Anaconda, July 2023. Web. <https://anaconda.com> (accessed on 01 August 2023)] in order to avoid dependency conflicts and allow the run using default parameters. Output files containing the genomic coordinates of predicted eccDNAs were then further processed using the program Intervene (version 0.6.5) [47] in order to identify intersecting eccDNAs across tested pipelines. For further studies on the profile of identified eccDNA, we selected all circles that were identified in at least three of the four pipelines, and identification in the cyrcular-calling pipeline was a mandatory criterion. This is due to the information on breakpoint sequences and gene overlaps provided by cyrcular-calling, but not by the other pipelines. Thus, the following Intervene data were combined in order to obtain all eccDNA identified by cyrcular-calling and at least any other two pipelines: cyrcular-calling + ecc_Finder + Flec + CReSIL; cyrcular-calling + ecc_Finder + Flec; cyrcular-calling + Flec + CReSIL; cyrcular-calling + ecc_Finder + CReSIL.

To evaluate the eccDNA profile in relation to chromosomal distribution, the number of eccDNA/mb/chr was calculated by dividing the number of eccDNAs in each chromosome by the size of each chromosome (in megabases) (chromosome size reference: Human Genome Assembly GRCh38.p14, available at https://www.ncbi.nlm.nih.gov/grc/human/data (accessed on 5 April 2024)). GC content of the sequence from 1000 bp upstream to 1000 bp downstream of the breakpoint was calculated using the GC content tool at the Jamie McGowan platform (https://jamiemcgowan.ie/bioinf/gc_content.html, accessed on 12 May 2024), and CpG Islands on the same region were identified using the CpG Islands tool at the Sequence Manipulation Suite platform (https://www.bioinformatics.org/sms2/cpg_islands.html, accessed on 30 May 2024), using the software’s standard calculation, which is performed with a 200 bp window moving through the sequence at 1 bp intervals (CpG island: region where the GC content is greater than 50% and the Observed/Expected value is greater than 0.6). Gene Ontology analysis was performed using The Gene Ontology Resource (https://geneontology.org/, accessed on 30 May 2024) (Annotation Version: GO Ontology database; DOI: 10.5281/zenodo.10536401. Released 17 January 2024). Intersection of eccDNAs identified in different samples was performed using the program Intervene, and intersection between genes carried by eccDNAs in different samples was performed using the Venn diagram webtool available at https://bioinformatics.psb.ugent.be/webtools/Venn/ (accessed on 3 April 2024).

Graphs for EV characterization; number of identified eccDNAs; eccDNA size and chromosomal distribution; eccDNA categorization; and gene fragment-containing eccDNAs were generated using the GraphPad Prism (version 10.2.0) software.

## 5. Conclusions

To the best of our knowledge, this constitutes the first study to demonstrate that human dermal fibroblast-derived extracellular vesicles carry eccDNA. It is also the first study to identify eccDNA in EVs using a long-read sequencing methodology. However, our findings suggest that there is no direct correlation between the genomic regions of eccDNAs identified in vesicles and in their cells of origin, nor is there consistency between different samples of EVs. Therefore, more extensive studies on the biogenesis of eccDNAs are merited. It is also appreciated that further exploration of EVs containing eccDNAs in different tissues will provide greater insights into the potential of the biomarker prospective of these molecules. Future implications for this novel research include using this framework method to elucidate factors and conditions that may influence the skin aging process and related biogenesis in human dermal cells.

## Figures and Tables

**Table 1:** Number of eccDNA molecules identified by pipelines cyrcular-calling, ecc_Finder, CReSIL, and Flec for each sample.

	Number of Reads (Top) Total Bases (Bottom)	Cyrcular-Calling	ecc_Finder	CReSIL	Flec	Common in All Pipelines	Common in Cyrcular- Calling and at Least Two Other Pipelines
EV1	1529 11,394,724	59	0	83	213	0	45
EV2	6945 57,736,046	251	136	504	907	107	227
EV3	5259 48,300,293	211	29	581	1725	18	150
D1	3836 30,999,272	24	0	1022	1427	0	18
D2	7194 49,947,289	295	64	407	694	58	246
D3	8855 66,141,179	250	113	352	603	80	214

## Data Availability

Raw sequencing files (fastq format) are available at the Sequence Read Archive (SRA, NCBI) under BioProject ID PRJNA1251131 (Biosamples accessions SAMN47998430, SAMN47998431, SAMN47998432, SAMN47998433, SAMN47998434, and SAMN47998435). Identified eccDNA regions are available as Supplementary Materials (Supplementary Document S1) to this manuscript. Any further data can be made available upon request from the authors.

## Supplementary Materials

The following supporting information can be downloaded at: https://www.mdpi.com/article/10.3390/ijms26094144/s1.

## Abbreviations

The following abbreviations are used in this manuscript:

eccDNA	Extrachromosomal Circular DNA
EV(s)	Extracellular Vesicles
NTA	Nanoparticle Tracking Analysis
SD	Standard Deviation
TEM	Transmission Electron Microscopy
