# Supplementary material for: Identification of eccDNA in Extracellular Vesicles Derived from Human Dermal Fibroblasts Through Nanopore Sequencing
Source: Int J Mol Sci. 2025 Apr 27;26(9):4144. doi: 10.3390/ijms26094144 (PMC12071958; doi:10.3390/ijms26094144)
Supplement: Supplementary file 1 [file ijms-26-04144-s001.zip › Supplementary Figure S1. Simonassi-Paiva et al. Re-submission. 16.04.25.pdf]

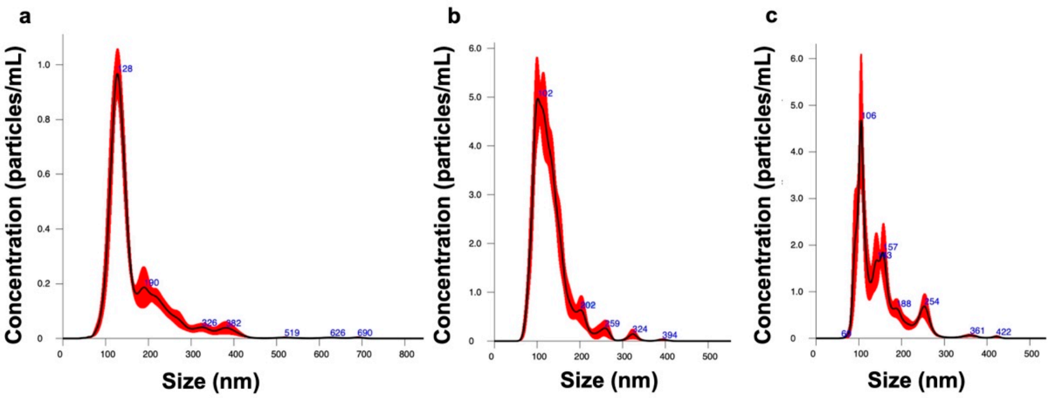


**Supplementary Figure 1**. Representation of concentration and size of EVs for each sample. EV1 (a); EV2 (b) and EV3 (c). Figures are generated by the Nanoparticle Tracking Analysis (NTA) system, where the y axis indicates particle concentration and the x axis indicates particle size. Each figure is generated from an average of five measurements performed on the same sample. Numbers in blue above the red peaks indicate nanoparticle size, and red peaks indicate sizes where there is a higher concentration of particles. Increased concentration peaks occurring between 100 nm and 200 nm in all samples are a positive indicator that these nanoparticles are extracellular vesicles.
