# Supplementary material for: Identification of eccDNA in Extracellular Vesicles Derived from Human Dermal Fibroblasts Through Nanopore Sequencing
Source: Int J Mol Sci. 2025 Apr 27;26(9):4144. doi: 10.3390/ijms26094144 (PMC12071958; doi:10.3390/ijms26094144)
Supplement: Supplementary file 1 [file ijms-26-04144-s001.zip › Supplementary Figure S2. Simonassi-Paiva et al. Re-submission. 16.04.25.pdf]

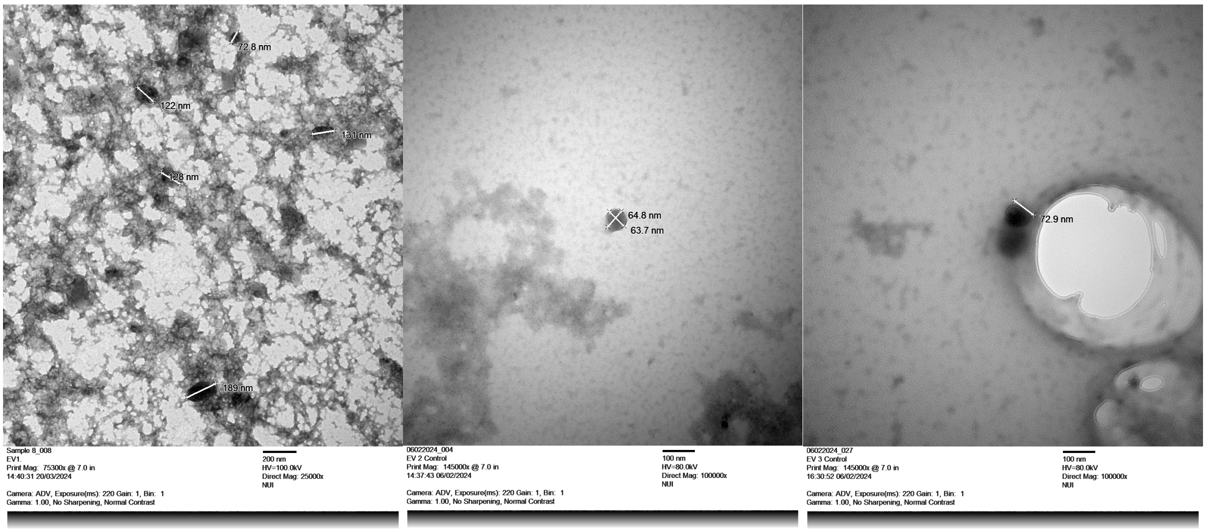

Figure S2. Uncropped TEM images. Extracellular vesicles observed under transmission electron microscopy. (Left) EV1. (Center) EV2. (Right) EV3.
