# Supplementary material for: Identification of eccDNA in Extracellular Vesicles Derived from Human Dermal Fibroblasts Through Nanopore Sequencing
Source: Int J Mol Sci. 2025 Apr 27;26(9):4144. doi: 10.3390/ijms26094144 (PMC12071958; doi:10.3390/ijms26094144)
Supplement: Supplementary file 1 [file ijms-26-04144-s001.zip › Supplementary Table S1. Simonassi-Paiva et al. Re-submission. 16.04.25.pdf]

Table S1. List of all unique genes overlapped by eccDNAs in each sample.

EV1	MUC19; VWA8; KCNK13; DPP10; SCN2A; TTN; DCDC2C; TOPBP1; TRPC1; EGFLAM; HCN1; MAP3K5; CAV2; KIAA1549; SKAP2; BPNT2; GRIA3
D1	RAB30; FRS2; DAD1; ADAMTS17; ZNF407; MBD5; GPD2; MRPS27; SSBP2; TPK1; PTPRD
EV2	SLC16A4; ACBD6; PCNX2; MTR; RIMS3; MAST2; AGBL4; DAB1; ROR1; LRRC7; ADGRL2; PRKACB; PLPPR5; ATRNL1; PDZD8; UPF2; SFMBT2; LRMDA; KCNMA1; KIF20B; CARD18; PKNOX2; LRRC4C; FAT3; CRY1; NAA25; ARID2; TESPA1; GRIP1; TPH2; TRHDE; NAV3; MGAT4C; CEP290; SIAH3; DACH1; GPC6; BRMS1L; MIPOL1; MNAT1; PTPN21; DPH6; FSIP1; UNC13C; THSD4; IREB2; AGBL1; CREBBP; RBFOX1; PCTP; PIEZO2; RTTN; FBXO15; PARD6G; PTPRM; FPR3; ZNF577; CCDC93; THSD7B; GRB14; COL3A1; PLCL1; WDR43; CTNNA2; RALGAPA2; RASSF2; BCAS1; ERG; CD200R1; TF; PLSCR5; KCNAB1; GHSR; TTC21A; ZNF619; ZNF620; TAFA1; FRMD4B; THUMPD3; COL8A1; FBXL5; RXFP1; TAPT1; GPM6A; KCNIP4; ANAPC4; SLC4A4; AREG; BMP2K; PAQR3; SGCD; ADAMTS12; ADAMTS16; ZSWIM6; ADCY2; AK9; NKAIN2; LAMA2; EYA4; SYNE1; ATXN1; QKI; RPS6KA2; GMDS; BMP5; ZNF292; FOXP2; CARD11; GNAI1; CFAP69; CASD1; SGCE; ASNS; COLEC10; CSMD1; ASPH; ZNF705G; NBN; GCNT1; GUCY2F; TENM1; DACH2
D2	TMIGD3; USH2A; NFIA; CLCA2; DPYD; MLLT10; KIAA1217; EPC1; KCNMA1; YAP1; PSMA1; EIF3M; CCDC73; DLG2; MTMR2; TAOK3; PCED1B; GRIP1; OSBPL8; SPATA13; CCDC169-SOHLH2,CCDC169; CCNA1; DIAPH3; DACH1; NPAS3; SLC25A21; MDGA2; FMN1; CDIN1; ANXA2; C15orf61; LONP2; PIEZO2; PTPRM; ANKRD12; RNF149; IL1RN; MGAT5; ARHGAP15; GTDC1; FMNL2; LRP2; METTL8; PARD3B; THADA; SRBD1; SOCS5; NRXN1; DNAH6; SLC24A3; CBLB; LSAMP; KALRN; RBMS3; GADL1; LZTFL1; FHIT; C3orf14; SYNPR; GRM7; PDZRN3; ROBO2; STAG1; PPP3CA; TBCK; ANK2; NDST3; USP38; SLC10A7; DCHS2; TMEM144; FNIP2; LDB2; SH3RF1; OCIAD2; TMEM165; CLOCK; EPHA5; KCNN2; BASP1; NADK2; ARL15; SLC38A9; CWC27; KIAA0825; DSE; SLC35F1; MAP7; PEX3; MPC1; AFDN; DOP1A; PGM3; SLC35B3; DUS4L-BCAP29,BCAP29; SND1; CHCHD3; CHN2; HERPUD2; CD36; ZFPM2; EXT1; IDO2; CHD7; SLC44A1; SVEP1; RABGAP1; NR6A1; TTC39B; ALDH1A1; MID2; NHS; DMD; APOOL; DACH2; FAM9B; DIAPH2
EV3	HRNR; RYR2; FMN2; DAB1; MINDY3; ZNF365; NCAM1; GALNT18; SPON1; NELL1; LRRC4C; ME3; CNTN5; C12orf42; RPH3A; CCDC91; CNTN1; PRICKLE1; NUP107; NALF1; CENPJ; DLEU7; CCDC198; SLC35F4; TMEM63C; CDH13; TCF4; RELCH; CCDC102B; PARD6G; SH3RF3; B3GALT1; NBEAL1; ANXA10; ERBB4; C2orf73; ALMS1; MACROD2; PCSK2; CLSTN2; PPM1L; FHIT; SUCLG2; INPP4B; GPM6A; NWD2; RHOH; EPHA5; COMMD10; MARCHF3; CHSY3; MFSD4B; REV3L; DSE; PEX7; GRM1; LHFPL3; EXOC4; ANKMY2; AUTS2; GNAI1; PCLO; RIMS2; ADCY8; MRPS28; ZNF704; GRIN3A; ELAVL2; NDP; CCNB3; DACH2
D3	ELAPOR1; SYT6; ATF6; NSL1; TGFB2; C1orf131; GCSAML; PLPP3; NEGR1; ATRNL1; MINDY3; MLLT10; AKAP13; ANKRD22; DDX10; GRIK4; ETS1; NTM; SOX6; C11orf58; TSG101; GAS2; TSPAN18; FCHSD2; PCDH9; SOX5; NAV3; NUDT4; ANKS1B; TNFSF13B; CDK8; FRY; KLHL1; RBM26; MDGA2; RTN1; DIO2; APBA2; FMN1; WDR72; REC114; CNTNAP4; CDH13; CRYBA1; ASIC2; B3GNTL1; PSMG2; CEP76; DLGAP1; PIGN; SMG9; GPD2; PLA2R1; METAP1D; FAM171B; HECW2; MAIP1; SPAG16; COL4A3; VPS54; EXOC6B; PTPRT; GRIK1; DSCAM; FBLN1; PLCXD2; PHLDB2; CNTN6; PCOLCE2; NAALADL2; CACNA1D; CADM2; NR3C2; MARCHF1; GABRA2; CCNG2; CDH18; WDR70; PLPP1; DEPDC1B; WDR41; FAM172A; PCSK1; NKAIN2; PHACTR1; PDE7B; PACRG; KIF6; ADGRB3; PLXNA4; CNTNAP2; ZPBP; CACNA2D1; CDK14; NSMCE2; TMEM71; NRG1; SMIM19; SPIDR; RAB2A; RUNX1T1; PAPPA; TTLL11; NTRK2; PTPRD; DOCK11; DMD; OPHN1; STS
